# Psychosocial Risk Factors, Interventions, and Comorbidity in Patients with Non-Specific Low Back Pain in Primary Care: Need for Comprehensive and Patient-Centered Care

**DOI:** 10.3389/fmed.2015.00073

**Published:** 2015-10-08

**Authors:** Aline Ramond-Roquin, Céline Bouton, Cyril Bègue, Audrey Petit, Yves Roquelaure, Jean-François Huez

**Affiliations:** ^1^Department of General Practice, University of Angers, L’Université Nantes Angers Le Mans, Angers, France; ^2^Laboratory of Ergonomics and Epidemiology in Occupational Health, University of Angers, L’Université Nantes Angers Le Mans, Angers, France; ^3^Department of General Practice, University of Nantes, L’Université Nantes Angers Le Mans, Nantes, France; ^4^Department of Occupational Health, University Hospital of Angers, Angers, France

**Keywords:** low back pain, primary care, risk factors, comorbidities, patient care management, psychosocial factors

## Abstract

Non-specific low back pain (LBP) affects many people and has major socio-economic consequences. Traditional therapeutic strategies, mainly focused on biomechanical factors, have had moderate and short-term impact. Certain psychosocial factors have been linked to poor prognosis of LBP and they are increasingly considered as promising targets for management of LBP. Primary health care providers (HCPs) are involved in most of the management of people with LBP and they are skilled in providing comprehensive care, including consideration of psychosocial dimensions. This review aims to discuss three pieces of recent research focusing on psychosocial issues in LBP patients in primary care. In the first systematic review, the patients’ or HCPs’ overall judgment about the likely evolution of LBP was the factor most strongly linked to poor outcome, with predictive validity similar to that of multidimensional scales. This result may be explained by the implicit aggregation of many prognostic factors underlying this judgment and suggests the relevance of considering the patients from biopsychosocial and longitudinal points of view. The second review showed that most of the interventions targeting psychosocial factors in LBP in primary care have to date focused on the cognitive-behavioral factors, resulting in little impact. It is unlikely that any intervention focusing on a single factor would ever fit the needs of most patients; interventions targeting determinants from several fields (mainly psychosocial, biomechanical, and occupational) may be more relevant. Should multiple stakeholders be involved in such interventions, enhanced interprofessional collaboration would be critical to ensure the delivery of coordinated care. Finally, in the third study, the prevalence of psychosocial comorbidity in chronic LBP patients was not found to be significantly higher than in other patients consulting in primary care. Rather than specifically screening for psychosocial conditions, this suggests taking into account any potential comorbidity in patients with chronic LBP, as in other patients. All these results support the adoption of a more comprehensive and patient-centered approach when dealing with patients with LBP in primary care. As this condition is illustrative of many situations encountered in primary care, the strategies proposed here may benefit most patients consulting in this setting.

## Introduction

Low back pain (LBP) refers to “pain and discomfort localized below the costal margin and above the inferior gluteal folds, with or without leg pain” (1). Non-specific LBP is defined as LBP that cannot be related to any specific disease, such as fracture, infection, osteoporosis, inflammatory or tumoral disease, or radicular syndromes (2). LBP during pregnancy is generally also excluded from this definition. Before discussing some recent developments in the field of psychosocial issues in patients suffering from non-specific LBP, this introduction briefly presents some epidemiological data, the current evidence regarding the different management strategies and the specific role of primary care in the management of these patients.

### Epidemiology

Low back pain is one of the most frequent complaints in the general population, generating pain, disability, and sometimes severe psychosocial consequences. An international review estimated the point prevalence and the 1-month prevalence of activity-limiting LBP lasting more than 1 day in the general population to be 11.9 and 23.2%, respectively (3). For most authors, acute LBP refers to LBP lasting for <6 weeks, sub-acute LBP to LBP lasting for 6–12 weeks, and chronic LBP to LBP lasting for more than 12 weeks (1, 2). Acute and sub-acute LBP are sometimes considered together, and named “(sub)acute.” After the onset of an episode of LBP, the probability of recovery decreases considerably when LBP persists beyond 6 or even 12 weeks (4).

Low back pain is a very frequent reason for practice visits. In Sweden, the 1-year prevalence of physician consultations for LBP was estimated to be 3.8% in the adult general population (5). In the US, the proportion of all physician consultations attributable to LBP was found to be about 2.3% in 2002 (6). LBP, and particularly chronic LBP, generates enormous costs for society, direct costs resulting from health care consumption, mainly health care providers (HCP) consultations, hospital admissions, medication, and medical imaging, and indirect costs relating to the large number of certificates for sick leave and the resulting work absenteeism (7–9).

Non-specific LBP belongs to the group of musculoskeletal disorders, which include diverse conditions affecting muscles, bones, and/or joints of the limbs or the spine. As for most other musculoskeletal disorders, non-specific LBP is multifactorial. Going beyond the initial models mainly focusing on biomechanical determinants, a biopsychosocial model of LBP integrating medical, occupational, psychosocial, and socio-demographic risk factors has been widely adopted by the health community in the last 20 years to reflect more accurately certain aspects of chronic pain and disability (10–12). Etiologic and prognostic factors need to be distinguished to implement relevant prevention and management strategies (13). Biomechanical factors seem to have a major impact on the occurrence of LBP episodes (14, 15), whereas psychosocial factors may have a greater influence on the persistence and on the impact of LBP (2). In particular, fear-avoidance beliefs, individual psychological problems, occupational context, and lack of social and familial support have generated growing interest during recent decades (16–18).

Patients with chronic LBP have been suggested to present particularly high prevalence of psychological, somatoform, and musculoskeletal comorbidity (9, 19, 20). The persistence of LBP may contribute to the development of these disorders (21, 22) and comorbidities have also been associated with poor outcome of LBP (17, 23). Moreover, patients with comorbidities present poorer physical and psychosocial functioning, poorer response to treatment, and higher rates of health care utilization than other patients suffering from LBP (24–28). Finally, physicians reported that psychiatric comorbidity made it more difficult to treat patients with chronic LBP and generated frustration (29). More generally, as for other complaints, an awareness of comorbidities is an important element for HCP when managing a patient with LBP. This is particularly true for primary care clinicians, who are “*accountable for addressing a large majority of personal health needs*, *developing a sustained partnership with patients*,” following the definition of Vanselow et al. (30).

### Management strategies and guidelines

Prevention of LBP has mainly been developed in occupational settings, due to the widely recognized role of work-related biomechanical and psychosocial risk factors of LBP and to its high prevalence in workers (31–33). Due to the recurrent character of LBP, most efforts have been devoted to reducing its impact with secondary or tertiary prevention (34). However, primary prevention remains particularly important and is widely recommended for exposed workers (13, 35–37). Given the relatively modest effects of traditional physical and organizational interventions, it seems necessary to develop integrated preventive action based on a participative approach (37–39).

Most therapeutic strategies for patients presenting with non-specific LBP have targeted medical and biomechanical determinants. In (sub)acute LBP, information, reassurance, advice to remain active, prescription of analgesics, exercises, and spinal manipulation are presented as options in different guidelines, whereas bed rest should be avoided (1, 40–42). Multidisciplinary interventions can also be proposed for certain workers presenting disability resulting from sub-acute LBP (41, 42). However, the effectiveness of these treatments is generally quite limited and most of the improvement observed is often attributable to the natural course of LBP (2). In chronic LBP, supervised exercises, cognitive-behavioral therapy (CBT), multidisciplinary treatment, and short-term use of analgesics for pain relief are commonly considered as evidence-based options, whereas back schools, spinal manipulation, use of antidepressants, acupuncture, and massage are more a subject of debate (21, 40–42). Physical therapies, such as heat, laser, ultrasound, or lumbar supports, and invasive treatments, such as steroid injections into the lower back, are generally not recommended (21, 41, 42). Surgery should only be considered in very specific situations (21, 41).

Following the acknowledgment of a biopsychosocial model of LBP, psychosocial risk factors have been considered more and more as promising targets for specific treatment (43, 44). Multidisciplinary programs addressing psychosocial factors in chronic LBP, mainly using an educational approach or CBT, have had a positive impact, but this has often been limited to short-term outcomes and few programs have been cost-effective (45–50). Furthermore, the wide and sustainable implementation of such programs presents a specific challenge, given the huge number of patients concerned. Psychosocial factors have recently also generated interest in earlier stages of LBP, and the guidelines invite HCPs to screen for and to take into account these factors in patients presenting with (sub)acute LBP (1, 35, 40–42).

### The specific context of primary care

The aim of this section is to reflect on the extent to which patients presenting with non-specific LBP also present similarities to other patients encountered in primary care, and thus on the extent to which research results related to this condition may be relevant to other situations primary HCPs face in their everyday clinical practice. Experiencing non-specific LBP does not systematically lead to contact with the health care system. In a Dutch study, only 30.6% of people with LBP consulted a family physician (FP) in a year (51). Many other symptoms, such as tiredness, cough, anxiety, abdominal pain or headache, that are among the more frequent reasons for consultation in primary care, share this characteristic, and it has been estimated that in general only one-third of people reporting any symptom actually consult a physician (52, 53). In a behavioral model, health care use has been suggested to be determined by predisposing factors (including demographic, socio-cultural factors, and health beliefs), enabling resources (personal and community resources), individual current perceived need, and past experiences of care, making the health behaviors “dynamic and recursive” (54). With regard to LBP, the level of disability and the co-existence of depression, which may influence the perceived need for consultation, are strongly associated with higher rates of health care utilization (7, 55, 56). In primary care, asking for and taking into account the motivation for a patient seeking help at this moment, whatever his symptoms, can contribute to the proposal of a relevant management strategy.

Medically unexplained symptoms (MUS) is the term used to cover a variety of common somatic symptoms whose etiology is unclear, alternatively called functional disorders, psychosomatic disorders, or somatoform disorders when symptoms persist (57). MUS have been estimated to affect between 19 and 35% of the population attending a FP (58, 59). Non-specific LBP represents one of the most frequent MUS in the general population and in primary care, along with pain in the limbs, dizziness, diarrhea, abdominal pain, fatigue, and headache (58, 60). Patients with MUS are more often dissatisfied with the treatment they receive from their FP than other patients (61). They sometimes develop specific strategies to legitimize their symptoms and to maintain medical attention (62). On the other hand, the context of medical uncertainty and the perceived lack of effective management strategies challenge most HCP, who often find patients with persistent MUS difficult to treat, especially when they are frequent attenders (63–67). FPs frequently report that these patients ask repeatedly for somatic investigations and treatment and that they have to prevent such inappropriate prescription, but it is not clear whether it is the patient or the HCP who generates such behaviors (68). In spite of their negative feelings, FPs consider that these patients should be managed in primary care, highlighting the major role of communication skills and the need for sustained, quality relationships (64, 69–71). This is totally in line with the European definition of family medicine, which is responsible for dealing with unselected complaints and “*with health problems in their physical, psychological, social, cultural and existential dimensions*,” developing a patient-centered approach and providing longitudinal continuity of care (72).

Primary care may be the setting in which implementing effective strategies for patients with LBP would be beneficial for the widest range of the population, since LBP constitutes one of the first reasons for consultation in this setting (73, 74) and FPs and physiotherapists are the professionals most frequently consulted (51, 55). Primary care may also be the setting in which consideration of the psychosocial factors would be the most relevant. Indeed, primary HCPs have often known patients with non-specific LBP for several years in their social, occupational, and family environments, and they are responsible for comprehensive and patient-centered care (72). However, FPs often feel frustrated when dealing with patients with non-specific LBP, and patients frequently share this feeling (29, 75, 76). FPs frequently report difficulties in adopting attitudes in accordance with the current recommendations and often find them to be irrelevant to the specific situations they face (77, 78).

A large part of the research related to psychosocial issues in patients with LBP has been undertaken in secondary or tertiary care settings. The external validity of these results in primary care might thus be questionable. In The Ecology of Medical Care published in 1961 then revised in 2001, it was estimated that each month 2% of patients consulting in primary care were referred to another physician, 4% were admitted to a hospital, and only 0.4% were admitted to a university medical center, from where most of the literature has originated (52, 53). Referral to specialized care or hospitalization does not occur randomly and the epidemiology is quite different between the different sectors of care. In primary care, non-specific LBP represents more than 70% of all the consultations for LBP, whereas serious diseases (fracture, infection, cancer, cauda equine syndrome, or inflammatory disease, considered altogether) are estimated to be found in <1% of the consultations in this context (79, 80). The natural course of LBP has also been found to be different depending on the sector of care being considered, with most of the patients from secondary care experiencing persistent bothersome pain, whereas a significant proportion of patients from primary care present fluctuating or improving pain (81).

Moreover, the methodological characteristics of specialized care-based research may not be totally relevant for primary care providers. Focusing on the most significantly disabled patients, with short-term follow-up, and with return-to-work being considered as the main outcome criterion, is reasonable from a specialized care perspective, but this appears to be insufficient from a primary care point of view. Based on specialized care-based research, it has long been considered that 90% of the patients with acute LBP recover in the first 6 weeks, with only 2–7% of them developing chronic LBP (1). In a recent review of prognostic studies, 65% of patients presenting with acute LBP in primary care still experienced pain 12 months after onset (82). These findings first look very dissimilar, but each represents a different facet of the same health problem. From a patient’s perspective, neither return-to-work nor pain constitutes an ideal outcome criterion if considered in isolation; patients’ recovery is a definitively more complicated and multidimensional construct (83). However, some outcome criteria seem to be more relevant than others, depending on the context in which the results of the research are intended to be used and implemented.

### Aims of this article

There is a great deal of literature about patients with non-specific LBP, but information related to those presenting in primary care is rarer. Some recent research has focused on the psychosocial issues related to the three main questions, yet these remained largely unanswered:

- Which psychosocial risk factors in patients consulting in primary care are associated with transition from (sub)acute to chronic non-specific LBP?
- Do interventions focusing on psychosocial factors improve the prognosis of patients consulting for (sub)acute non-specific LBP in primary care?
- Do patients consulting for non-specific chronic LBP more often present psychosocial comorbidities than patients consulting for other reasons in a primary care setting?

The aims of this article are to summarize these recent findings and to discuss certain perspectives regarding implementation of future strategies in order to meet the challenge of non-specific LBP in primary care, strategies that may be relevant for many other patients encountered in this setting.

## Psychosocial Risk Factors for Transition from (Sub)Acute to Chronic Non-Specific LBP in Primary Care

In 2003, the Cochrane Back Review Group stated that highlighting factors influencing the outcome of (sub)acute LBP was a major challenge to improving patient prognosis (84). Indeed, the identification of such factors may help HCPs to evaluate the prognosis of individual patients and adapt their management strategies. As stated above, psychosocial factors have been found to be of particular importance in the transition from (sub)acute to chronic LBP. A huge amount of epidemiological research has been conducted on this topic in the last 20 years in a wide range of settings and with different methods. This has resulted in inconsistent or even contradictory findings, which have required context- and objective-oriented selection and rigorous methodological evaluation. A systematic review was therefore undertaken in 2010 to answer the specific question: which psychosocial factors are associated with transition from (sub)acute to chronic non-specific LBP in primary health care? (85).

Briefly, this review identified 18 relevant cohort studies, in which 16 different psychosocial factors belonging to the socio-economic field, the occupational field, the psychological field, or the cognitive and behavioral field had been explored for their prognostic value. Most of the socio-economic factors, as well as job satisfaction, anxiety and pain control were found to be rarely associated with the outcome of LBP. The results were inconsistent for compensation issues, whereas evidence was still not found regarding social support and somatization. Depression, psychological distress, passive coping strategies, and high levels of pain-related fear were found to be predictive of poor evolution of LBP in some of the studies which explored their prognostic value, but the predictive ability was modest. Finally, the factors most strongly linked with subsequent evolution were self-perceived general health (including physical and psychosocial dimensions), patients’ expectations of recovery and the care provider’s perception of risk of persistence at baseline.

Despite a fairly substantial amount of available literature, prediction of the evolution of patients consulting for (sub)acute LBP in primary care on the basis of assessment of any potential psychosocial factors remains challenging. The methodological heterogeneity of the studies included in the review is not sufficient to account for the inconsistency of the results observed. Any single factor might explain only a very small fraction of the variability observed in any cohort, although it can be of major importance in certain individual histories. Several authors have suggested systematically considering combinations of prognostic factors in order to predict the evolution of LBP and have proposed multidimensional scales, including evaluation of psychosocial factors, work-related factors, pain-related factors, and limitations due to LBP (86, 87). However, these scales have not been proved to perform better than subjective, overall assessment of the risk of chronic LBP by the HCP (88). Indeed, another review found that a patient’s recovery expectations were among the strongest and the most consistent predictors of work-related outcomes in patients presenting with non-specific (sub)acute LBP in various settings (89). This is fully congruent with what we consider as being the main finding of this review, namely, the significant predictive ability of patients’ or providers’ overall judgments about the likely evolution of LBP.

This latter finding is not surprising. Subjective, overall judgment, by the patient or by the HCP, implicitly aggregates many potential individual prognostic factors. Non-specific LBP may result from and interact with various problems, ranging from mainly biomedical problems to essentially psychosocial issues, originating from a continuum of mixed situations (75). Moreover, this condition may be related to individual (and even intimate) difficulties, and/or to collective issues (for example, related to occupational, societal, or cultural characteristics) (90, 91). These determinants often vary from one period of time to another for a given patient. Many other conditions are affected by such a variable combination of multidimensional determinants, especially in the field of chronic pain and functional syndromes (92–94). Even the evolution of chronic diseases, such as diabetes, chronic obstructive pulmonary disease, and chronic heart failure, traditionally considered as mainly somatic, is affected by various factors, including psychosocial issues (95–97). Characterization of any health problem presented by any patient at any time according to these two dimensions, i.e., biomedical/psychosocial and individual/collective, is essential to enable HCPs to support the patient in coping with his/her individual situation in his/her unique context.

Moreover, the HCP’s approach should take account of both the current symptoms and the history of previous similar complaints. Repeated episodes of LBP should not be seen as independent events, but rather as recurrent symptoms related to a single (and evolutive) health condition (81, 98). Traditional epidemiological methods often consider episodes independently and at best take into account the number of previous episodes. However, LBP is frequently recurrent or persistent, with fluctuating disability, increasing during acute flare-ups (99). The classical distinction between acute, sub-acute, and chronic LBP is not sufficient to describe its overall evolution, and epidemiologists still lack tools to interpret this type of non-linear evolution, although certain authors have tried to standardize the definitions of recurrence (100). The development of life-course epidemiology may help to explore these issues in a more relevant way, although it is still unlikely to embrace the whole complexity of the evolution of LBP (101). Beyond these methodological considerations, HCPs should consider both the immediate determinant(s) of the current episode (such as a physical effort) and the general vulnerability factors (whether these are biomedical, psychological, or social) underlying the whole history of LBP. This is an important (and expected) skill of primary care providers, which in fact applies to most chronic conditions, such as diabetes and asthma, where the HCP must focus both on the overall disease-related condition and on flare-ups.

In conclusion, psychosocial factors should undoubtedly be considered in addition to other factors from a longitudinal point of view, in order to predict the risk of poor evolution of LBP. In primary care settings, the subjective and overall assessment by the HCP (providing that the latter knows the patient well enough) may constitute the most relevant prognostic tool, performing as well as any currently available multidimensional scale and being easier to use. Such a conclusion may be quite specific to primary care settings, for two main reasons. First, patients consulting in primary care are less selected than those seen in specialized contexts and may present a wider range of determinants of the transition to chronic LBP. This might, therefore, reduce the predictive ability of any formal scale or algorithm, which is always limited in the number of risk factors taken into account. Second, a long-term relationship with the patient and a patient-centered and comprehensive perspective of care, both characteristics of primary care, are probably key elements in the reliability of the HCP’s judgment.

## Interventions Focusing on Psychosocial Risk Factors in Non-Specific (Sub)Acute LBP in Primary Care

Consistent with clinical observations and epidemiological findings about the association between psychosocial factors and transition to chronic LBP, the guidelines recommend screening for potential psychosocial factors when LBP persists after several weeks, and to address them, with no further detail (1, 35, 40–42). Addressing the psychosocial issues is sometimes difficult for HCPs when the patients consult them explicitly for LBP. Some FPs have mentioned their perceived lack of relevant strategies, whereas others seem to be afraid of damaging their relationship with such patients when exploring potential psychosocial issues (76). Some interventional research has been conducted on this topic in various settings in recent years, with inconsistent methodological quality and leading to contrasting results. A systematic review was therefore conducted in 2013, aiming to identify and critically evaluate the effectiveness of any interventions targeting psychosocial factors in adults presenting with non-specific (sub)acute LBP in primary care (102).

Briefly, 13 clinical trials were included in this review, in which most interventions aimed to modify health beliefs and improve coping skills. Information strategies were the most frequently implemented interventions, with high quality evidence of no effectiveness in terms of most of the patient-centered criteria. Cognitive-behavioral therapies yielded very low quality evidence of moderate effectiveness in terms of pain, function, quality of life, work issues and health care use. There was little evidence regarding the three other types of strategy identified, namely individual education, group education, and work coordination.

It seems that research has to date failed to identify any intervention focusing on psychosocial factors that would lead to significantly better outcomes for patients presenting with non-specific (sub)acute LBP in primary care settings. No specific and well-defined intervention can, therefore, be currently recommended to HCPs in this context. This conclusion contrasts with the widely acknowledged biopsychosocial model. Several hypotheses have been proposed to explain the lack of effectiveness of a promising intervention consisting of systematic screening of psychosocial factors and a minimal intervention strategy in LBP patients by general practitioners in a primary care setting (103). GPs in the intervention group actually adopted a less biomedical attitude, but identification of psychosocial factors and the impact of the minimal intervention strategy remained suboptimal (104). This may partly have been due to insufficient training of GPs in using standardized screening tools or in delivering interventions targeting psychosocial factors (104, 105). Similarly, the four dimensional symptoms questionnaire has been shown to be a feasible and valid way for GPs to screen for psychological disorders in sick-leave patients in the context of primary care (106), but its usefulness to support minimal intervention aiming at improving the prognosis of distressed patients has not been demonstrated to date and may require substantial GP training (107, 108). Regardless of the screening tool or the intervention content, some authors have argued for subgrouping the patients and delivering targeted interventions only to those considered at high risk of chronicity and who may benefit most (109). However, such subgrouping has, to date, not resulted in a greater impact of these interventions (110, 111). More generally, recent literature is fairly convergent in acknowledging the lack of efficacy of the current strategies targeting psychosocial factors (112, 113). In fact, it seems unlikely that any standardized intervention focusing on a single (or even a delimited set of) psychosocial factor(s) would ever fit the needs of most patients in a primary care setting, where they present heterogeneous situations involving an infinite number of determinants.

The exact nature and role of psychosocial factors in the evolution of LBP should probably be questioned. Following traditional epidemiological models, all the factors identified have to date been considered as being causally and independently linked to the evolution of LBP. However, some might be causal determinants of poor evolution, whereas others might only be indicators of increased risk. Moreover, some determinants may have a proximal role and others a more distal role, the latter influencing previous determinants to a greater or lesser extent. Interventional research has mainly focused on cognitive and behavioral factors, especially fear-avoidance beliefs and coping skills. By contrast, HCPs have often identified more private and contextualized psychosocial issues, such as marital conflict, financial difficulties, or work-related litigation, often in combination rather than in isolation, as being determinants in the evolution of their patients suffering from LBP (76). Cognitive and behavioral factors may be largely determined by certain other factors, including (among others) previous history of LBP, other previous illness experiences, socio-cultural aspects, and also what we called above “private and contextualized psychosocial issues.” The strategies to measure and to address the latter are much more difficult to design and to implement than those related to cognitive and behavioral factors, because they vary considerably from one person to another, and because they often involve intimate dimensions. However, considering only cognitive and behavioral factors constitutes a threatening reductionism of the biopsychosocial model that has underlain their identification. Stewart et al. published an interesting paper questioning the current ideas and clinical applications related to psychosocial factors (114). They deplored the narrowness and superficiality of the way the biopsychosocial model has been understood and applied in most research works and guidelines. They deservedly argued for paying more attention to “*the underlying reasons for a person’s behavior or thoughts*,” and for taking into account “*the individual’s unique experience and meaning of pain*.” Indeed, also addressing potential distal determinants could result in a positive impact when managing patients with non-specific LBP.

Going one step further, care should probably be taken not to reduce the biopsychosocial model to its single psychosocial dimension. Only three studies in our review had implemented multi-component interventions, including several of the following: physical, informational, behavioral, and occupational components (115–117). These studies did not demonstrate any clinically relevant influence on patients’ evolution, but their low number, certain methodological shortcomings, and the wide differences between the interventions implemented to prevent any firm conclusion. However, the only study, which assessed the relative impact of each component, considering them in isolation then in combination, suggested that, for short-term follow-up outcomes at least, “the effect was greatest when the interventions were combined” (116). In the specific field of occupational LBP, the literature suggests that interventions also taking into account the occupational environment have been more effective than those focusing only on physical rehabilitation to prevent long-term disability (118–121). Simultaneously targeting several determinants in multi-component interventions, including in particular biomechanical, psychosocial, and occupational factors, may result in greater impact, provided that each of these components is relevant to the patient’s situation and that the whole strategy is sufficiently coordinated.

As with many other conditions in primary care which are multifactorial and impact on various domains (functional, psychological, social, occupational, etc.), patients with non-specific LBP often consult several HCPs. FPs, physiotherapists, occupational physicians (OPs), and osteopaths are the most frequently involved in the management of these patients, with less frequent interventions of rheumatologists, orthopedists, psychiatrists, neurologists, pain specialists, psychologists, radiologists, acupuncturists, etc. (55, 122). This constitutes a challenge in terms of coordination of care. HCPs from different disciplines or professions, and sometimes from different generations or cultures, often have different representations of the medical condition and of the most appropriate way to manage the patient. HCPs’ beliefs and attitudes seem to influence those of their patients and their clinical management (123). Guidelines for the management of non-specific LBP state that “it is important that information and treatment are consistent across professions, and that all health care providers closely collaborate with each other,” since divergence between different HCPs’ attitudes and strategies might contribute to poor outcome of LBP (1, 37).

Coordination of care has been defined as “the deliberate organization of patient care activities between two or more participants (including the patient) involved in a patient’s care to facilitate the appropriate delivery of health care services” (124). It is also intended to avoid the wasteful duplication of diagnostic or therapeutic procedures and to reduce the risk of conflicting medications and advice (125). Care coordination largely consists of interprofessional exchanges and collaborations, which involve shared responsibility and decision-making, respectful partnership, interdependency, and symmetry of power relationships in a dynamic and interactive process (126, 127). The previously mentioned behavioral model of health care use has been adapted in order to be applied to the coordination behaviors of HCPs (124). In this adaptation, care coordination is a function of predisposing factors (motivation of HCPs to coordinate care, depending on individual attitudes and knowledge but largely determined by professional background and training), enabling resources (such as shared information systems, use of protocols, quality of relationships with other HCPs, and specific payment policies), and the perceived need for coordination (in our case, a patient with a complex and multifactorial situation requiring the involvement of many stakeholders). Such a model explicitly acknowledges the role of pre-existing HCP-related factors that are not only easily altered but also highlights professional and/or organizational strategies that have the potential to foster interprofessional collaboration.

In occupational LBP, frequent interactions and enhanced collaboration between the different HCPs involved, and between HCPs, workers, colleagues, and supervisors have resulted in better outcomes for workers (128, 129). Similarly, interdisciplinary coordination has often been the critical feature of multidisciplinary rehabilitation programs aiming to prevent long-term sick leave related to various conditions (130). Specific collaboration between FPs and OPs is especially important in the case of non-specific LBP, as for other conditions resulting in occupational disability. Both FPs and OPs acknowledge that increased collaboration probably results in better quality of care for patients (131). But such collaboration is often limited, mainly due to the apparently differing interests and lack of interactions favored by the independent organization of the health care system and occupational settings. Practical difficulties, lack of knowledge about their respective roles, and negative representations from FPs have also been reported (132, 133). Joint interdisciplinary training programs and standardized cooperation protocols have been proposed to improve such collaboration (134, 135). However, evidence is still needed to establish the effectiveness of these strategies (134, 136).

Care coordination constitutes one of the core tasks of primary care providers because of their specific skills and their easy, frequent, and trustful contacts with the patient (30). This is particularly true in countries where they act as gatekeepers to specialist care (137). Some patients with persistent non-specific LBP may benefit from tailored multi-component interventions, involving different stakeholders. In such cases, enhanced interprofessional collaboration and better care coordination, mainly driven by primary HCPs, may result in better outcomes (138). Indeed, the grouping of relevant and complementary skills should be steered toward a common objective: addressing all the needs of the patient, rather than dividing them into their individual dimensions.

## Psychosocial Comorbidities in Patients with Non-Specific Chronic LBP in Primary Care

As stated above, LBP and comorbidities interact with each other and together influence health care-seeking behavior and health-related quality of life. Improved knowledge of frequent comorbidities in the patients presenting with chronic non-specific LBP may support primary HCPs in adapting their management strategy. Epidemiological research has been consistent in showing that these patients present high levels of prevalence of psychological, somatoform, and musculoskeletal comorbidity (19, 20, 139, 140). However, this literature has most often reported on the comorbidity of patients who were not representative of those consulting with LBP in primary care, and has compared it with results from the general population rather than with other patients from clinical settings. Moreover, the use of questionnaires in cross-sectional or retrospective designs has often resulted in a high level of risk of participation, recall, and declaration bias. It has therefore not been clear whether the prevalence of such comorbidity is specifically higher in the patients presenting with chronic non-specific LBP in primary care than in other patients consulting in this setting. Finally, there is little information available about social comorbidities in patients with chronic LBP. A recent epidemiological study (141) investigated the prevalence of psychological, social, somatoform, and musculoskeletal health problems presented to their FPs by patients with chronic non-specific LBP, compared to patients consulting in the same setting without LBP, using longitudinal data from a primary care practice-based research network with long experience (59, 142) (Box 1).

Box 1 **Methodological characteristics of study focusing on psychosocial comorbidities in patients with chronic LBP in primary care (141).**

Design:

Case-control study embedded in a historical cohort.

Setting:

The Dutch Transition Project database: a primary care practice-based research network, currently of nine GPs working in four different practices with about 15,000 patients, with long experience of data collection.

Data collection:

Routine and prospective coding of all patients’ consultations using the International Classification of Primary Care (ICPC), a standardized classification coding the reason(s) for encounters, the diagnosis(es), and the intervention(s) in each consultation.

An episode of care includes any consultations (one or several), which are related to the same health problem in an individual. The duration of the episode is the time between the first and the last consultation for the health problem being considered and its title is the diagnosis considered by the GP most accurately to describe the patient’s condition in the last consultation, whether it be a disease, a syndrome or a symptom.

Patients:

- Cases: patients older than 18 years, diagnosed with one episode of non-specific low back pain (code L03 in the ICPC) lasting for 90 days or more between 1996 and 2013.
- Controls: selected from consulting patients who had never been diagnosed with an episode of non-specific LBP.
- Cases and controls were matched 1:1 for gender, age, practice of listing, and date of consultation.

Data analysis:

Seven groups of codes from the ICPC were considered specifically:

- Three ICPC chapters: musculoskeletal (excluding low back pain), psychological, and social.
- Four clusters of somatoform symptoms: cardiorespiratory symptoms, gastrointestinal symptoms, musculoskeletal symptoms, and general symptoms.

The prevalence of these health problems was compared between the cases and the controls, during each of these periods independently: the year before the beginning of the episode of low back pain, the first year after it and the second year after it, using conditional logistic regression.

In total, 1511 patients who had presented with an episode of chronic non-specific LBP in 4 different FP practices and their 1511 matched controls were included. Mean age at the beginning of the episode was 51 years, and 60% were women. The median duration of each episode of chronic LBP was 2.1 years. Compared to their controls, the patients with chronic LBP significantly more often presented musculoskeletal problems (in addition to LBP). Unexpectedly, they presented similar levels of prevalence in terms of psychological, social, and non-musculoskeletal somatoform comorbidities.

These original findings contrast with most results available in the literature. However, two studies investigating the prevalence of certain frequent comorbidities in patients presenting to their FP with LBP, compared to other patients consulting in this setting, found only very weak associations between non-specific LBP and depressive or anxiety disorders (5, 143). The prevalence of psychosocial and somatoform comorbidity might be high in specific subgroups of patients suffering from severe chronic LBP rather than in more unselected populations of patients such as those seen in primary care (8). Moreover, it is likely that patients consulting in a primary care setting present higher levels of prevalence of comorbidity (including psychosocial and somatoform problems) than the general population. This illustrates the specific need for collecting data from the sector of care where the patients are thought to benefit from the research results, and hence the relevance of data collected through primary care practice-based research networks (144).

This study showed that patients presenting with chronic non-specific LBP in primary care actually suffered from certain psychosocial problems and somatoform disorders but did not support the view that they present these problems more often than other patients consulting in this setting. Beyond these quantitative results related to the prevalence in groups of patients, it is important to consider the individual impact of comorbidity. Rather than systematically screening for certain comorbidities in the specific population of patients presenting with chronic LBP, we suggest that primary HCPs should remain aware of any comorbid condition, including those in the psychosocial field, when managing those patients, as they should do for any other patients presenting with any (especially chronic) condition. HCPs should explore and address all potential factors with patients (whether considered as a risk factor or as a comorbidity) that might interact with LBP and impact on their functioning and quality of life.

The patient-centered clinical method may provide HCPs, and particularly primary HCPs, with a useful framework to guide their management strategy (145). Although definition of patient-centeredness is still debated in the literature, several components are consistent between different authors: considering the individual disease and illness experience; taking into account, the whole person from a biopsychosocial point of view; finding common ground in understanding what the problem is and mutually agreeing on a management strategy; enhancing patient–clinician relationships (146, 147). In comparison with usual care, adoption of a patient-centered approach has resulted in lower pain and less psychological distress in patients presenting with chronic musculoskeletal pain in primary care, as well as in diverse positive outcomes in other groups of patients (148, 149). This clinical method seems highly relevant to progressive identification and discussion with the patient of the potential determinants of his/her persistent pain, reasons for seeking care, current priorities, and main expectations in his/her individual specific context. These determinants, motivations, priorities, and expectations should be considered iteratively and from a longitudinal point of view, because they frequently change over time.

Patient centeredness is strikingly complementary to care coordination, as discussed in the previous section. Care coordination may be considered as a process which needs to be implemented within a patient-centered approach finally to produce integrated care, defined as “patient care that is coordinated across professionals, facilities, and support systems; continuous over time and between visits; tailored to the patients’ needs and preferences; and based on shared responsibility between patient and caregivers for optimizing health” (150, 151). Continuity of care, which represents the patient’s experience of the integration of care, has been linked to higher patient satisfaction and lower undesired health care utilization in a variety of different patients and settings (152). Continuity is particularly valued by patients suffering from chronic conditions and appears to be a critical element in the management of any multifactorial disorder (153).

## Conclusion and Perspectives

These recent findings could be summarized by stating that research has to date (1) failed to identify any combination of well-defined psychosocial risk factors, which would reliably predict the risk of transition from (sub)acute to chronic non-specific LBP in primary care; (2) failed to identify any intervention focusing on psychosocial factors, which would significantly reduce this risk in a cohort of patients; and (3) failed to identify any increased prevalence of psychosocial comorbidity in patients with chronic non-specific LBP, compared to other patients consulting in primary care. However, such a summary would be incomplete. Research has also shown that primary care providers are often able to make an accurate judgment about the likely evolution of an episode of non-specific LBP and to identify psychosocial issues in their patients. However, the best way to transpose these skills into effective clinical strategies has yet to be found.

First of all, it is important to bear in mind that delivering such comprehensive intervention including a psychosocial dimension to any patient consulting for non-specific LBP in primary care would probably be uselessly costly, irrelevant, and may even constitute harmful overtreatment in some cases. Such a strategy should probably mainly target patients presenting with sub-acute LBP. At this stage, the persistence of pain often coexists with psychosocial issues, such as psychological distress, cognitive modifications, familial problems, work-related difficulties, or social isolation. Whether the persistent pain results in or is the result of these psychosocial issues – the reality is probably a mix of those, and different from one patient to another – the latter should be considered because they are part of the problem and they contribute to poor outcomes (21, 154). Over the same period (sub-acute LBP), patients may also become discouraged with regard to the limited effectiveness of medical treatment to alleviate their symptoms, and to the low value of further medical investigations. Finally, support from their socio-familial and occupational environment sometimes decreases after the acute stage of the condition (155). Primary care providers are very often involved in the management of patients facing this type of challenging situation, not only related to non-specific LBP but also to pain in any location, and more generally to any disabling but not life-threatening condition. This constitutes a sensitive period during which HCPs should widen their focus beyond the strict biomedical aspects. We propose that this attitude should also be applied to patients presenting with more recent but recurrent symptoms, or with chronic symptoms but recently unusual repeated consultations. These different scenarios often reflect evolving situations, from a stable state – with or without symptoms – to another state with poor(er) adaptation, leading to seeking help from the health care system.

**Figure 1:**
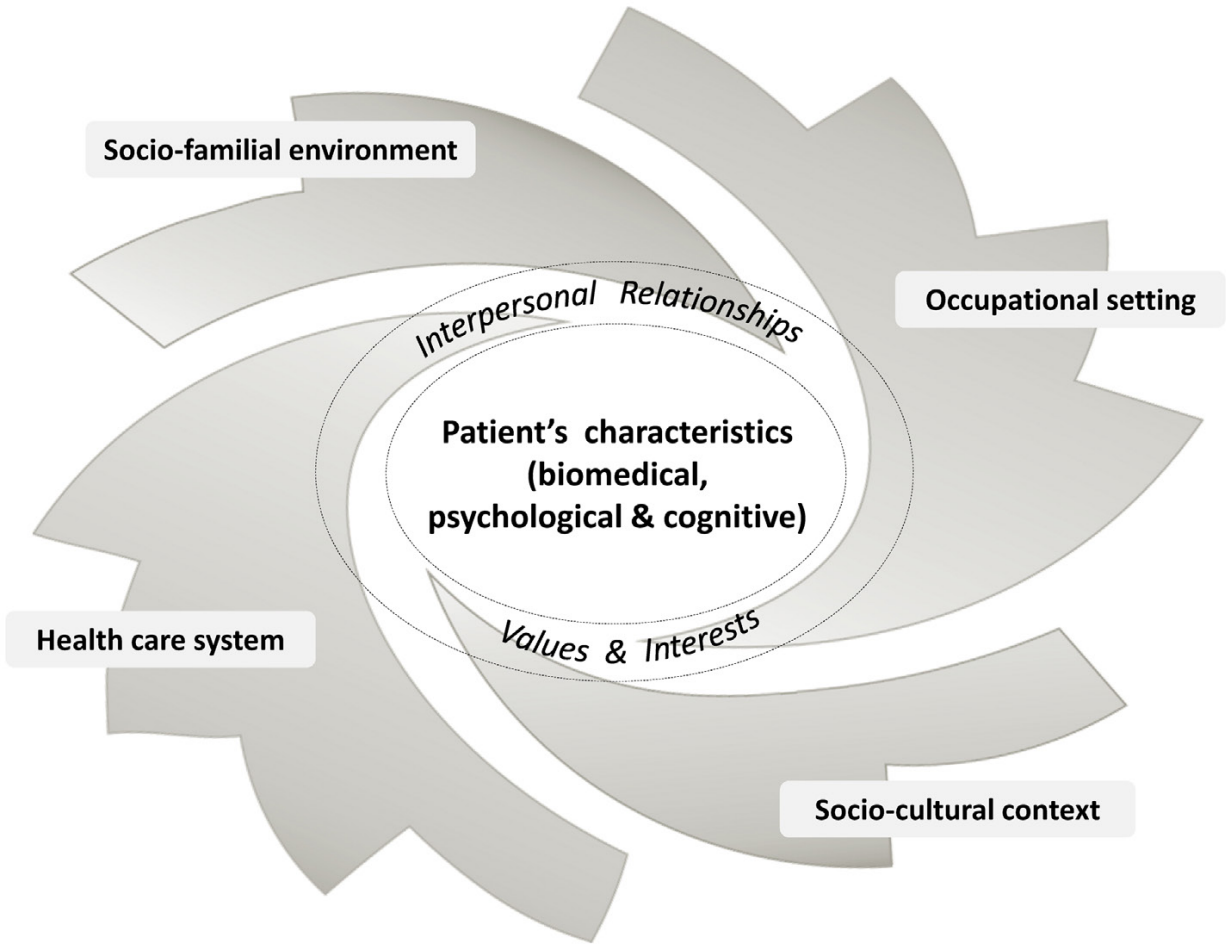
**Patient with low back pain in his/her biopsychosocial environment**.

**Figure 2:**
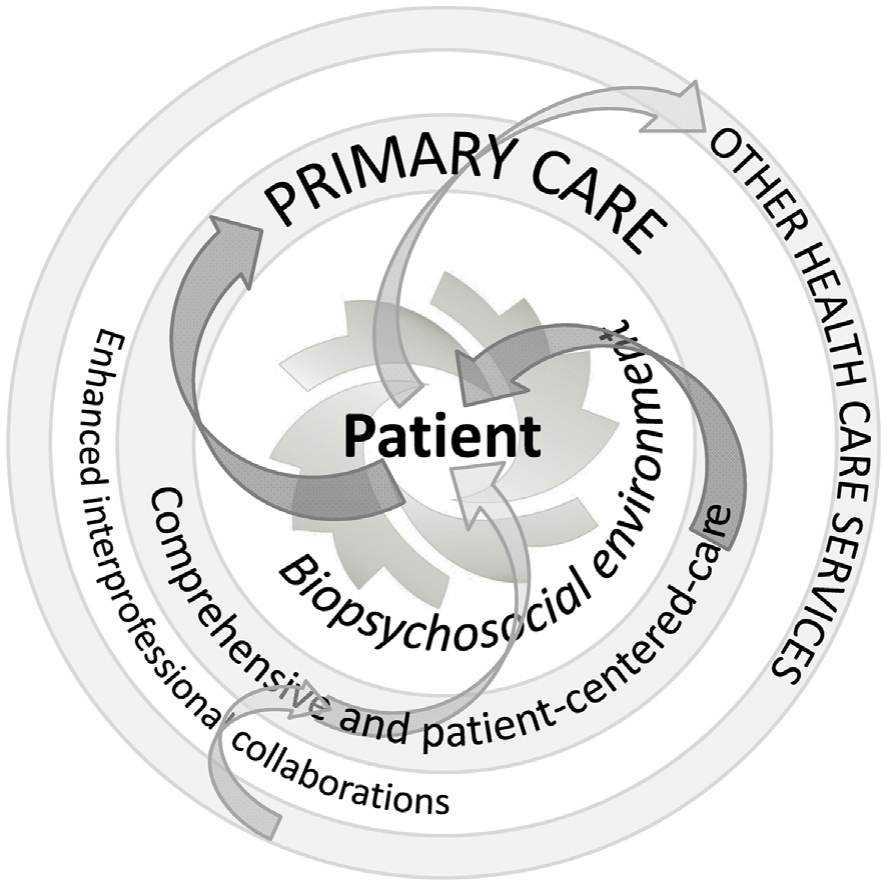
**Suggested principles of management for patients with low back pain**.

With regard to the care strategy itself, any intervention should follow a progressive and recursive approach that cannot be uniform or limited *a priori*. After the initial steps mainly focusing on screening for serious disease and symptom relief, primary HCPs should go beyond the current musculoskeletal complaints. They should consider the whole person from a wider and long-term point of view, including any biopsychosocial issues, from risk factors to motivation and from comorbidities to expectations. They should try to identify the potential obstacles to improvement in the environments of their patients and also their potential resources, which are often undervalued although they might constitute a very interesting lever in better coping with their conditions (156–158). Primary HCPs are often aware of certain psychosocial issues, but too often limit their field of interest and intervention to the strictly biomedical field. They should be concerned by all these dimensions and explore and address in greater depth those which seem to interact with the patients’ complaints (Figure 1). HCPs should also invite patients progressively to embrace this perspective. This may help them to find common ground and achieve some agreement on the different needs of the patient and on the most relevant care strategy over time, in line with a person-centered approach. Depending on each specific situation, the intervention may sometimes consist of several (simultaneous or successive) components involving different stakeholders. In such a case, primary HCPs should be aware that continuity of care is a critical point, and, with the patient’s agreement, should engage in enhanced interprofessional collaboration and coordination of care (Figure 2).

The next step would be to develop a pragmatic guidance tool following these principles, intended for clinicians managing patients presenting with persistent or recurrent non-specific LBP and supporting them in a wider approach that would be consistent with the theoretical biopsychosocial model (159). This tool should be adaptable, according both to the characteristics of the patient and the context in terms of the socio-cultural environment and health care system resources, to ensure broad and sustained utilization. Its development and experimental implementation should follow a stepwise and iterative approach, to improve its relevance and acceptability for both clinicians and patients, such as processes followed when designing complex interventions for other health issues in primary care (160, 161). Its effectiveness should be assessed throughout this process, using pragmatic study designs and mixed methods, and following realistic evaluation principles, rather than isolated randomized clinical trials which do not determine what works, for whom and in which context (160, 162).

We of course acknowledge that adopting such an approach will not cure all the patients in primary care who suffer from non-specific LBP, but we hypothesize that it may contribute to significant improvement of their prognosis and quality of life in cases of persistent or recurrent symptoms, by helping patients to cope with them better. Given that this strategy is not specific to the lower back, such an approach might be relevant for patients with other types of musculoskeletal pain, and be in part generalized to other patients consulting in primary care.

## Conflict of Interest Statement

The authors declare that the research was conducted in the absence of any commercial or financial relationships that could be construed as a potential conflict of interest.
